# Chronic Expanding Hematoma Complicated by Life Threatening Hemoptysis and Respiratory Arrest—A Case Report

**DOI:** 10.1002/rcr2.70665

**Published:** 2026-06-30

**Authors:** Anthony Shing‐yiu Yu, Chung Yin Cheung, Chor Ting Ng, Chi Ching Chan, Ying Fung Lo, Judianna Suet Ying Yu, Wallace Hon Cheung Fan, Alwin Wai Tak Yeung

**Affiliations:** ^1^ Department of Medicine and Geriatrics Ruttonjee and Tang Shiu Kin Hospitals Hong Kong China

**Keywords:** bronchopleural fistula, chronic expanding hematoma, hemoptysis

## Abstract

Chronic expanding hematoma (CEH) is a rare, indolent entity mostly linked to prior tuberculosis, thoracic surgery or trauma, with gradual long‐term enlargement and potentially fatal complications. We present a 73‐year‐old Nepalese male without classic predisposing risk factors, presenting with mild hemoptysis in 2017 and a large right pleural hematoma on computed tomography (CT). He declined surgical evacuation and later developed progressive hematoma expansion and subsequent life‐threatening hemoptysis complicated by type 2 respiratory failure and suspected bronchopleural fistula in 2025. He was deemed inoperable, failed conservative management and succumbed to nosocomial pneumonia. Post‐mortem histology confirmed CEH with dense fibrous tissue and organized blood clot. This case highlights early recognition and surgical intervention as critical for CEH, even in patients without antecedent thoracic insult.

## Graphical abstract

In this report, we describe a patient with the rare diagnosis of chronic expanding hematoma, leading to life‐threatening hemoptysis and ultimately type 2 respiratory failure. This case highlights the importance of proactive management for CEH due to its potentially fatal complications.

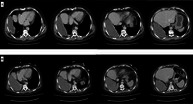

## 1 Introduction

Chronic expanding hematoma (CEH), a rare clinical entity within thoracic medicine, is defined as a slowly enlarging hematoma that progresses in size over a period of years. The proposed pathogenesis involves recurrent cycles of microhemorrhage, local inflammation, pathological neovascularization and tissue organization within capillaries of granulation tissue, leading to progressive hematoma enlargement and the formation of a thick fibrous capsule over time [1]. Thoracic CEH is disproportionately reported in Asian populations, with the vast majority of cases occurring as a long‐term sequela of prior pulmonary tuberculosis, intrathoracic surgical intervention, blunt or penetrating thoracic trauma [2]. Given its nonspecific clinical and radiological presentation, patients typically undergo extensive diagnostic evaluation to rule out more common differential diagnoses, including complicated parapneumonic effusion, empyema, malignant pleural mesothelioma, and metastatic pleural disease, prior to establishing a diagnosis of CEH [2]. The clinical course of thoracic CEH is indolent, with gradual expansion occurring over several years before the onset of severe or life‐threatening complications [2].

Diagnosis of thoracic CEH poses considerable clinical challenges, as it requires a high index of suspicion coupled with correlating clinical history and radiological findings. The diagnostic gold standard remains histopathological confirmation of an organized hematoma encapsulated by a thick fibrous capsule following surgical excision [1]. On computed tomography (CT) imaging, thoracic CEH typically demonstrates heterogeneous soft tissue density with a thickened, often partially calcified capsule, distinguishing it from simple pleural effusions or unorganized hematomas [3].

We present a rare case of thoracic CEH in a 73‐year‐old Nepalese male with no identifiable history of pulmonary tuberculosis, thoracic surgery, or traumatic thoracic injury, who subsequently developed life‐threatening hemoptysis and suspected bronchopleural fistula, culminating in respiratory failure. This case underscores the critical importance of early clinical recognition and proactive multidisciplinary management of thoracic CEH given the potential to develop fatal complications.

## 2 Case Report

A 73‐year‐old Nepalese man with a medical history of well‐controlled asthma and hypertension presented to our institution with mild, intermittent hemoptysis in 2017. Contrast‐enhanced thoracic CT demonstrated a large loculated right pleural soft tissue collection measuring 13.7 × 6.5 × 7 cm, consistent with an organized pleural hematoma (Figure 1). There was no history of pulmonary tuberculosis, cardiothoracic surgery or thoracic trauma. Ultrasound‐guided thoracentesis yielded altered, old blood, with no evidence of acute haemorrhage. Pleural fluid cultures were negative for acid‐fast bacilli and bacteria, and cytology was negative for malignancy. Given the patient's mild clinical symptoms and stability following oral tranexamic acid, he declined surgical evacuation of the organized pleural clot. A repeat thoracic CT in 2020, performed following an episode of recurrent hemoptysis, identified new gas pockets within the pleural hematoma in the absence of any intervening pleural procedures and infections, raising clinical suspicion for early bronchopleural communication (Figure 2A).

**FIGURE 1:**
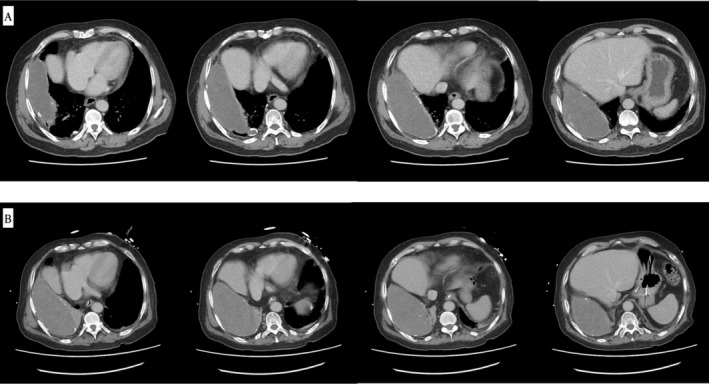
(A) CT Thorax in 2017 showing a pleural hematoma measuring 13.7 × 6.5 × 7 cm. (B) CT Thorax in 2025 showing slight increase in size of pleural hematoma measuring 14.0 × 7.8 × 8.4 cm.

**FIGURE 2:**
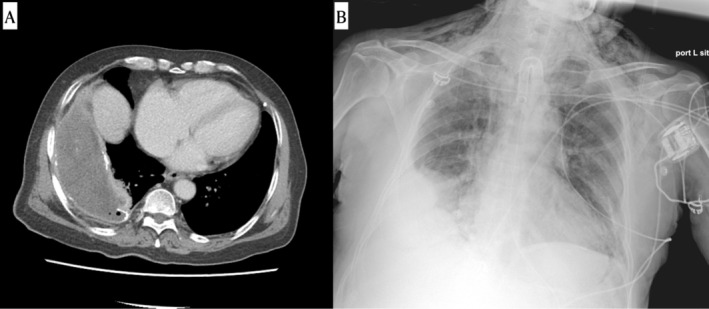
(A) CT Thorax in 2020 showing a loculated gas pocket within the pleural hematoma, implying a bronchopleural fistula. (B) Surgical emphysema due to positive pressure ventilation.

The patient subsequently returned to Nepal and was lost to follow‐up for 5 years, re‐presenting to our hospital in late 2025 with chest pain and uncontrolled hypertension. On admission, his blood pressure was 185/100 mmHg. Physical examination revealed bilateral expiratory wheezing and reduced air entry across the right hemithorax. New‐onset atrial fibrillation was also detected. Urgent CT aortography excluded acute aortic dissection as a cause of chest pain but demonstrated interval enlargement of the right pleural hematoma to 14.0 × 7.8 × 8.4 cm, with increased peripheral dystrophic calcification (Figure 1B). Following adequate blood pressure control, oral Apixaban was initiated for stroke prophylaxis in the setting of newly diagnosed atrial fibrillation.

Shortly thereafter, the patient developed life‐threatening hemoptysis complicated by type 2 respiratory failure, requiring emergent endotracheal intubation and mechanical ventilation in the Intensive Care Unit. Apixaban was immediately discontinued. Bronchoscopy via the endotracheal tube revealed blood oozing from the right lower lobe bronchus, corresponding to the anatomical location of the pleural hematoma. Endotracheal aspirate analysis via the BioFire FilmArray respiratory panel identified *Klebsiella pneumoniae* and methicillin‐resistant *Staphylococcus aureus* (MRSA), prompting targeted antimicrobial therapy with levofloxacin and vancomycin. CT bronchial arteriography demonstrated no abnormally dilated or hypertrophied bronchial arteries as a source of haemorrhage. Thoracic CT again identified intralesional gas pockets but did not clearly delineate a direct anatomical communication between the hematoma and the bronchial tree.

A clinical diagnosis of life‐threatening hemoptysis secondary to thoracic CEH was established. Cardiothoracic surgery consultation was obtained for surgical evacuation and capsulectomy. However, the patient was deemed inoperable due to persistent type 2 respiratory failure, restricted lung volumes due to hematoma size and frailty. Thoracic ultrasound revealed poor diaphragmatic excursion. Ultrasound‐guided percutaneous aspiration of the hematoma was attempted but no fluid could be aspirated, as the clot was densely organized. The patient's clinical course was further complicated by subcutaneous emphysema of the chest and neck, attributed to positive pressure ventilation exacerbating air leak via the bronchopleural fistula (Figure 2B). This was managed conservatively and resolved without the need for intercostal chest drain insertion. The patient failed multiple attempts for ventilator weaning due to recurrent hemoptysis and progressive decompensated type 2 respiratory failure, requiring elective tracheostomy for long‐term ventilatory support.

Despite supportive care, including frequent bronchoscopic toileting and suctioning for recurrent hemoptysis, the patient developed nosocomial pneumonia and succumbed to respiratory failure 2 months following tracheostomy. Post‐mortem histopathological examination of the pleural lesion showed dense fibrous tissue with dystrophic calcification, patchy chronic inflammatory infiltrate, mature fibroadipose tissue and organized blood clots, consistent with the diagnosis of a CEH (Figure 3). No histological evidence of tuberculosis, malignancy or acute infection was identified.

**FIGURE 3:**
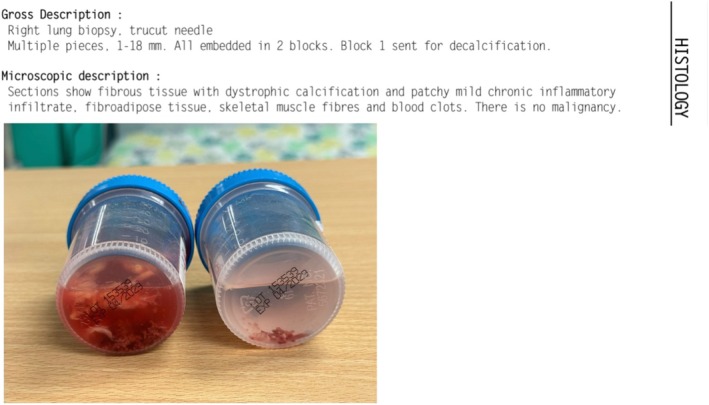
Post‐mortem biopsy consistent with pleural hematoma.

## 3 Discussion

Chronic expanding thoracic hematoma was first characterized in 1980, and since its initial description, it remains an extremely rare clinical entity, with most available evidence derived from isolated case reports and small case series [1]. Notably, nearly all reported cases of thoracic CEH occur in patients of Asian descent, with classic predisposing factors including prior tuberculosis, thoracic surgery, or trauma [4]. Clinical presentation is typically nonspecific, with most patients reporting gradual onset of chest discomfort, progressive dyspnoea or mild hemoptysis, correlating with slow hematoma expansion over years [2]. Radiologically, thoracic CEH is distinguished by a heterogeneous pleural mass with a thick, variably calcified fibrous capsule on CT imaging, a hallmark finding that aids in differentiating CEH from other pleural pathologies [3]. The progressive enlargement of CEH is postulated to arise from a self‐perpetuating cycle: irritant effects of blood breakdown products within the hematoma induce recurrent microhaemorrhage from neovascularized capillaries in the fibrous capsule, leading to ongoing hematoma expansion and tissue organization [5].

Thoracic CEH carries significant clinical risk, with life‐threatening hemoptysis and sudden mortality documented in the literature [2]. Surgical excision of the hematoma and its fibrous capsule is regarded as the first‐line and definitive treatment modality, although intraoperative massive haemorrhage was a reported complication. Preoperative selective arterial embolization may be considered to reduce perioperative bleeding risk in high‐risk cases [6]. A Japanese case series of five patients with thoracic CEH who underwent pleuropneumonectomy or total capsulectomy reported successful hospital discharge, with variable intraoperative blood loss ranging from 905 to 6590 mL [6]. For patients with unorganized hematomas or those unfit for major surgery, repeated therapeutic thoracentesis has been proposed as a palliative alternative, although its efficacy is limited in patients with densely organized clots, as in our case [7].

This case represents a rare presentation of thoracic CEH, given the absence of classic predisposing risk factors such as prior tuberculosis, thoracic surgery or trauma. The patient's recurrent hemoptysis localized to the right lower lobe by bronchoscopy correlated directly with the anatomical location of the CEH, and clinical and radiological findings were consistent with a secondary bronchopleural fistula. Positive pressure ventilation likely exacerbated air leak via the bronchopleural fistula leading to subcutaneous emphysema. Additionally, the patient's refractory type 2 respiratory failure and inability to wean from mechanical ventilation were postulated to result from the impaired respiratory mechanics secondary to mass effect from the large CEH, as well as reduced diaphragmatic excursion. Surgical resection, the gold standard treatment, was not feasible due to the patient's frailty and end‐stage respiratory failure, and percutaneous hematoma aspiration was unsuccessful due to dense clot organization. The diagnosis of CEH was ultimately confirmed on post‐mortem histopathology.

In conclusion, thoracic CEH should be included in the differential diagnosis for patients with a persistent, slowly enlarging pleural mass on CT imaging, especially after malignancy and active infection have been excluded. Key radiological features include a heterogeneous encapsulated mass with peripheral calcification. Management of thoracic CEH is complex and requires a multidisciplinary approach involving pulmonologists, cardiothoracic surgeons, and radiologists, with early surgical intervention recommended to prevent life‐threatening complications.

## Author Contributions

Anthony Shing‐yiu Yu and Chung Yin Cheung prepared all the figures and wrote the manuscript. Chor Ting Ng, Chi Ching Chan and Ying Fung Lo performed the post‐mortem biopsy. Judianna Suet Ying Yu, Wallace Hon Cheung Fan and Alwin Wai Tak Yeung contributed to the discussion and editing of the manuscript. All the authors reviewed the manuscript.

## Consent

The authors declare that written informed consent was obtained for the publication of this manuscript and accompanying images using the consent form provided by the Journal.

## Conflicts of Interest

The authors declare no conflicts of interest.

## Data Availability

The data that support the findings of this study are available from the corresponding author upon reasonable request.
